# Ligand-based adoptive T cell targeting CA125 in ovarian cancer

**DOI:** 10.1186/s12967-023-04271-8

**Published:** 2023-09-05

**Authors:** Haihong Zhao, Lina Wu, Jiemin Dai, Ke Sun, Zhenguo Zi, Junhua Guan, Liwen Zhang

**Affiliations:** 1 Department of Obstetrics and Gynecology, The Fifth People’s Hospital of Shanghai, Fudan University, Shanghai, 200240 China; 2 School of Pharmacy, Shanghai Jiao Tong University, Shanghai, 200240 China; 3 Shanghai Institute of Immunology, Department of Immunology and Microbiology, Shanghai Jiao Tong University School of Medicine, Shanghai, 200025 China

**Keywords:** Chimeric antigen receptor (CAR), Chimeric receptor (CR), CA125, Mesothelin, Ovarian cancer

## Abstract

**Background:**

Ovarian cancer (OC) is a highly aggressive gynecological malignancy prevalent worldwide. Most OC cases are typically diagnosed at advanced stages, which has led to a 5-year overall survival rate of less than 35% following conventional treatment. Furthermore, immune checkpoint inhibitor therapy has shown limited efficacy in the treatment of patients with OC, and CAR-T therapy has also demonstrated modest results owing to inadequate T cell infiltration. Therefore, novel strategies must be developed to enhance T cell persistence and trafficking within the OC tumor microenvironment.

**Methods:**

In this study, we developed a novel adoptive T-cell therapy for ovarian cancer based on a chimeric antigen receptor structure. We used a ligand-receptor binding motif to enhance the therapeutic effect of targeting CA125. Since mesothelin can naturally bind to CA125 with high affinity, we concatenated the core-binding fragment of mesothelin with the 4-1BB and CD3ζ signal fragments to assemble a novel CA125-targeting chimeric receptor (CR). The CAR structure targeting CA125 derived from the 4H11 antibody was also constructed. CR- and CAR-encoding RNA were electroporated into T cells to evaluate their antitumor activity both in vitro and in vivo.

**Results:**

While CR-T or CAR-T cells exhibited moderate activity against two ovarian cancer cell lines, T cells co-expressing CR and CAR exhibited a superior killing effect compared to T cells expressing either CR or CAR alone. Furthermore, upon interaction with ovarian tumors, the ability of CR and CAR T cells to release activation markers and functional cytokines increased significantly. Similarly, CR and CAR co-expressing T cells persistently controlled the growth of transplanted ovarian cancer tumors in NSG mice and significantly prolonged the overall survival of tumor-challenged mice. Transcriptome sequencing revealed that the survival and cytotoxicity of T cells co-expressing CR and CAR were significantly altered compared with those of T cells expressing either CR or CAR.

**Conclusion:**

Our findings demonstrate that CA125 targeting CR and CAR can synergistically kill ovarian cancer cells, indicating that CA125 targeting by the two binding motifs simultaneously in tumors may improve the therapeutic outcomes of ovarian cancer treatment.

**Supplementary Information:**

The online version contains supplementary material available at 10.1186/s12967-023-04271-8.

## Background

Ovarian cancer (OC) is a fatal gynecological disease with the highest mortality rate among gynecological malignant tumors. Owing to its indolent properties and lack of effective screening tools, over 75% of ovarian cancers are diagnosed at advanced stages, which leads to a poor 5-year overall survival of less than 35% [1]. Chemotherapy based on cisplatin or carboplatin, which is the conventional treatment for ovarian cancer, remains the first-line treatment. However, tumor recurrence is common in advanced ovarian cancer after chemotherapy [2]. Highly aggressive cancers are often considered immunologically “cold” tumors. Although various immune checkpoint inhibitors (ICIs) and cytokine therapies are gradually being used in clinical applications, their therapeutic effects have not improved significantly [3].

In recent years, chimeric antigen receptor T cell (CAR-T) therapy has demonstrated a transformative clinical performance against hematological malignancies [4]. The findings from studies on this therapeutic method have helped develop a revolutionized strategy for solid tumor treatment. Recently, several CAR-T therapies for ovarian cancer have been explored, such as FOLR1 [5, 6], MSLN [7], TAG-72, and CD47 [8]. However, research progress on the use of CAR-T for ovarian cancer is yet to make a breakthrough; conversely, the neurotoxicity and systemic side effects of CAR-T cells have been reported in multiple instances [9, 10]. Based on preclinical evidence, CAR-T cells need to exhibit moderate affinity for the targeting protein to prevent T cell dysfunction [11, 12]. Therefore, fine-tuning the affinity of the CAR structure to the target can be a promising strategy to improve clinical response. The common CAR structure is a specific antibody scFv domain linked to an immunoreceptor tyrosine-based activation motif (ITAM). However, scFv affinity is often laboratory-selected without accuracy or rationale [13]. Alternatively, partial HGF fragments can bind to MET expressed on tumor cells with high specificity, indicating the potential for effectively targeting malignant mesotheliomas using ligand-directed CAR T-cell therapy [14]. Therefore, further investigation is warranted to determine whether natural ligand-receptor interactions can be used in designing CAR for ovarian cancer treatment.

Both mesothelin and CA125 are overexpressed in approximately 88% of ovarian cancer cases. CA125 is a highly glycosylated mucin comprising a large cleaved and released domain and a conserved domain [15]. 4H11 is a specific hybridoma-generated antibody targeting the conserved domain of CA125, which has been used in CAR-T therapy and tested in a phase I clinical trial [16]. Additionally, according to multiple reports, the glycosylphosphatidylinositol-anchored glycoprotein mesothelin binds to CA125 with strong binding kinetics, facilitating the peritoneal metastasis of ovarian tumors [17–20]. Glycosylated and non-glycosylated mesothelin can bind to the 156-amino-acid region of membrane-expressed CA125 [18]. In contrast, 64 amino acids at the N-terminus of mesothelin (region 296–359) are the minimum fragment that completes binding to CA125 [21]. In this study, we genetically fused this sequence with 4-1BB and CD3ζ signal fragments to generate a novel structure named chimeric receptor (CR). We also obtained the scFv sequence from a CA125-specific antibody (4H11) [22] and constructed the CA125-targeting CAR structure for comparison. Here, we attempted to systematically examine the antitumor activity of our engineered CR- or CAR-expressing T cells targeting ovarian tumor cell lines, which could help us develop an alternative approach for ovarian cancer treatment.

## Methods

### Mice and cell lines

Six-week-old immuno-deficient NSG female mice were purchased and bred under pathogen-free conditions at the Shanghai Model Organisms Center for Cancer Research. Mice were maintained under 12:12 h light: dark cycles at 60% humidity and 21–25 °C. All experiments were performed according to the instructions of the National Institutes of Health and the Institutional Animal Care and Use Committee.

Mycoplasma-free SKOV3, OVCAR3, and U251 tumor cells were obtained from the ATCC cell bank with authenticated STR profiling. The cells were cultured in RPMI 1640 (Gibco) supplemented with 10% heat-inactivated FBS and 100 U/mL penicillin/streptomycin sulfate. All cells were transduced with firefly luciferase-GFP (LucG) lentiviruses and sorted on a Bio-Rad S3e cell sorter to obtain 100% transduced populations.

T cells were isolated from peripheral blood mononuclear cells (PBMCs) using Ficoll density gradient centrifugation and cultured in X-VIVO 15 medium (Lonza) supplemented with 100 U/mL IL-2 (R&D Systems). T cells were activated according to a previously reported protocol [23]. Briefly, isolated T cells were mixed with Dynabeads Human T-Activator CD3/CD28 (Gibco) at a 1:3 ratio and cultured in IL-2-supplemented X-VIVO 15 medium. Dynabeads were removed using DynaMag™-2 Grate (Gibco) after 5 days, and activated T cells were cultured and supplied with an IL-2-supplemented medium to maintain T cell growth. When the T cells expanded and their size declined to approximately 350 μm^3^ (normally 15–20 days after activation), they were considered ready for transformation or cryopreserved for further use.

### Engineered T-cell production

The CA125-binding sequences of CR and CAR are shown in Additional file 8: Table S1. Partial mesothelin and 4H11 scFv DNA fragments following the T7 promoter were synthesized (Sangon Biotech) and separately cloned into the L482A RNA expression plasmid backbone. All constructs contained a CD8 hinge and transmembrane domain, 4-1BB costimulatory domain, and CD3ζ signaling domain with a 150 bp poly-A cassette. The two plasmids were transcribed in vitro into RNA using a capped RNA Synthesis Kit (Hongene Biotech) according to the manufacturer’s instructions. The synthesized RNAs were stored in a refrigerator at − 80 °C.

1 × 10^7^ prepared T cells were mixed with 5 μg of synthesized CR, CAR, or CR/CAR equally mixed mRNA in 100 μL of opti-MEM medium (Gibco), transferred into a 0.2 cm Gene Pulser Cuvette, and electroporated using a BTX ECM830 instrument at 500 V for 0.7 ms [24]. After electroporation, the T cells were immediately transferred into pre-warmed T cell culture medium and cultured in a cell incubator for 18–24 h.

### qPCR and western blotting

Total RNA was extracted using the RNAeasy Mini Kit (QIAGEN), followed by cDNA synthesis using the Hifair II 1st strand cDNA Synthesis Kit (Yeasen Biotech). Quantitative real-time PCR was performed using Hieff qPCR SYBR Green Master Mix (Yeasen Biotech). The primers for CR and CAR detection are listed in Additional file 8: Table S1.

Whole-cell lysates of SKOV3, OVCAR3, and U251 cell lines for western blotting were prepared in 200 mL of RIPA buffer (Beyotime Biotech) supplemented with a protease inhibitor cocktail (Roche). The samples were incubated on ice for 10 min and subjected to BCA analysis. Total protein (20 mg) was used for the western blot analysis. The primary antibodies used in this study were anti-MUC16 antibody (Abcam) and anti-β-actin antibody (Abcam). Goat anti-rabbit-IgG-HRP (Abcam) was used as the secondary antibody. 

### Cytotoxicity assay

CR-T cells, CAR-T cells, CR + CAR-T cells, and NTD cells were washed twice with IL-2-free medium and co-cultured with SKOV3-lucG, OVCAR3-lucG, and U251-lucG cells at gradient E:T ratios in 96-well flat-bottom plates separately in triplicate for each sample. The plate was then placed in an IncuCyte S3 dynamic imaging and analysis system to detect GFP fluorescence. The change in mean GFP fluorescence intensity was detected every 4 h and monitored in real-time for 72 h. The killing efficiency was calculated as follows: Killing efficiency = (total fluorescence area − residual fluorescence area)/total fluorescence area × 100%.

### Flow cytometry

The transfection efficiency of CR and CAR in each T cell group was assessed by staining with an Fc-CA125 full-length protein (ACROBiosystem) and PE-Fc antibody (eBioscience). CA125 expression in tumor cells was detected using a rabbit anti-human CA125 antibody (Abcam) and FITC-donkey-anti-rabbit antibody (eBioscience). After the cells were co-cultured with tumor cells for 24 h, the activation markers CD69-PE and CD25-APC (eBioscience) were detected on the T cells. CD1107A-PE (eBioscience) expression on T cells was detected after co-culture with tumor cells for 4 h in a Golgi-stop solution (BD Biosciences). All T cells were gated on CD3-BV421- and CD8-AF700 (eBioscience)-positive cell populations. Flow cytometric analysis was performed using an Attune NxT V6 flow cytometer (Thermo Fisher). The acquired data were analyzed using FlowJo version ten software (Tree Star).

### ELISA and diluted ELISpot assay

For ELISA, 1 × 10^5^ effector cells per well were stimulated with 1 × 10^5^ tumor cells for 24 h. IL-2 and IFN-γ present in the supernatant were detected using DuoSet ELISA Development Systems (R&D Systems), according to the manufacturer’s instructions. For the diluted ELISpot assay, 1 × 10^3^ effector cells per well were stimulated with 1 × 10^5^ tumor cells for 16–20 h in duplicate or triplicate, and the spots were visualized with a Human IFN-γ-precoated ELISPOT kit (DAKEWE) according to the manufacturer’s instructions. The plates were scanned and analyzed using an ELISpot Reader (Mabtech).

### Xenograft assay

SKOV3-LucG cells were harvested under healthy conditions and filtered using a 100 μm mesh. The cells were then washed with PBS, and 3e6 cells suspended in 100 μL of PBS were injected into the right flank of NSG mice. On day 14, the mice in each group received tail vein injections of 5e6 T cells in 100 C of PBS. Tumor progression was monitored every 2 or 3 days using a Vernier caliper and weekly by bioluminescence signaling using an IVIS imaging system (PerkinElmer), followed by the intraperitoneal injection of D-luciferin (30 mg/mL). The body weight and survival of mice were monitored every 2 or 3 days for 30 days after adoptive T cell treatment.

### Bulk RNA-seq

CR, CAR, CR + CAR, and NTD T cells (1e6) were co-cultured with SKOV3 cells for 24 h in triplicate. Following this, the co-cultured cells were labeled using a CD45-PE antibody (BioLegend) for 15 min at room temperature and sorted using an EasySep™ Human PE Positive Selection Kit II (Stemcell). Briefly, CD45-PE-stained cells were mixed with a PE selection cocktail and RapidSpheres™. CD45-PE-positive cells were labeled with magnetic beads and sorted using a magnet. The sorting efficiency was assessed using an FASC greater than 95%. Total RNA was extracted from the sorted cells using the RNAeasy Mini Kit (QIAGEN), and RNA was detected with integrity and deep sequenced by Novogene, Beijing. The functional analyses of DEGs were performed using DAVID and KEGG for Ingenuity canonical pathway enrichment. The heatmaps and volcano plots of differentially expressed genes in each group and gene set enrichment analysis (GSEA) results were depicted using R (version 3.6.3).

### Statistical methods

Analyses were performed using GraphPad Prism 8 (version 8.0.1). Data are presented as mean ± SEM. Data from each group were compared using a two-tailed unpaired Student’s t-test, based on the type of data. Significance was considered at P < 0.05 (*P < 0.05, **P < 0.01, ***P < 0.001, and ****P < 0.0001); no significance was considered at P > 0.05. Multiple comparison corrections were used for experiments involving multiple groups, as indicated in the figure legends.

## Results

### Identification of CA125-targeting CAR and CR-T cells

**Fig. 1:**
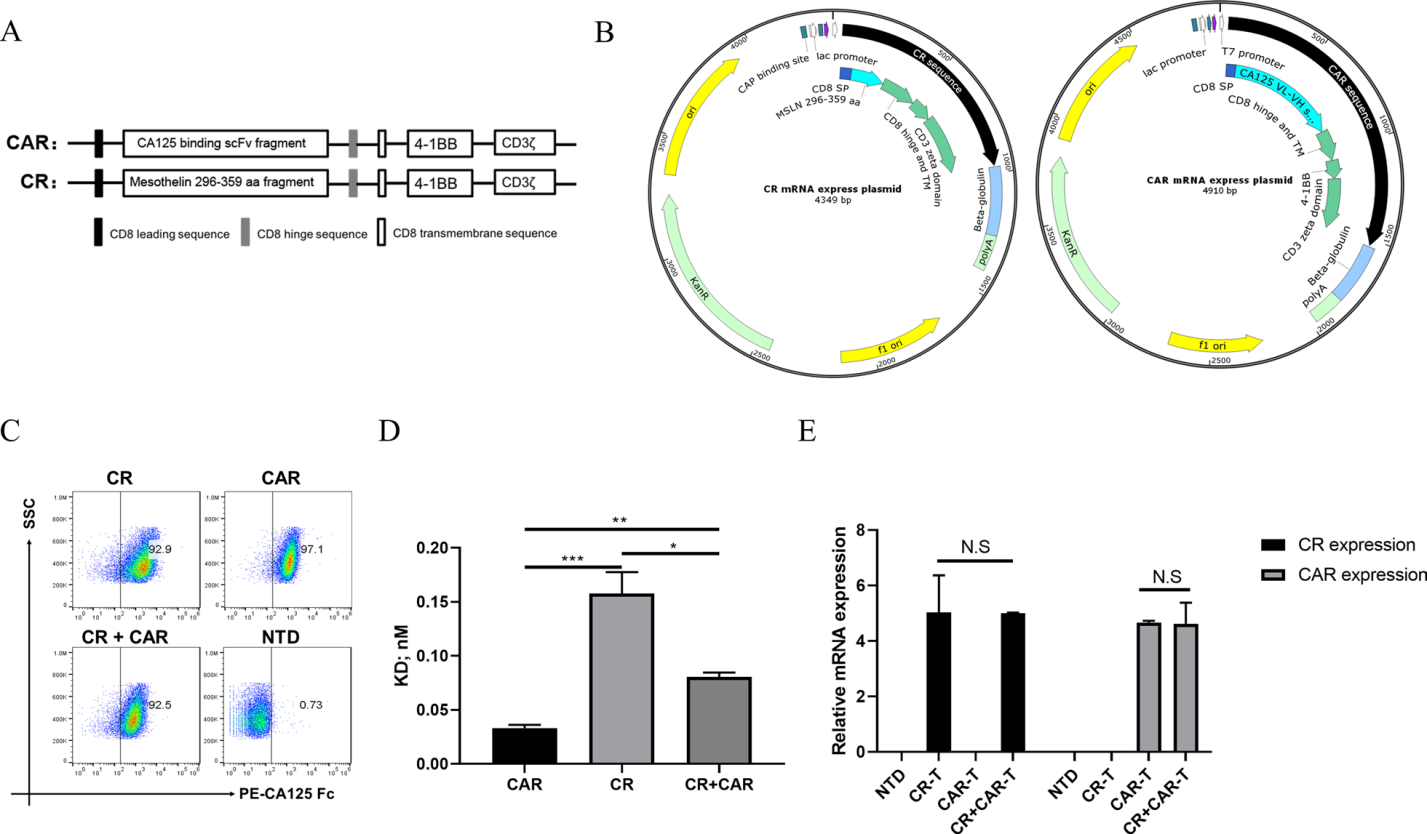
Construction and detection of CR and CAR expressed T cells. **A** The original structure of chimeric antigen receptor (CAR) and chimeric receptor (CR) targeting CA125; **B** the structure of CR and CAR-expressing plasmids; **C** flow cytometry detection of the CA125 binding activity of T cells transduced with CR, CAR, and CR + CAR; **D** the Kd value of CR, CAR and CR + CAR comparation targeting CA125 protein. **E** qPCR results of mRNA expression levels of electro-transduced CR, CAR, and CR + CAR expressed T cells group after 24 h. All data are presented as the mean ± SEM of triplicate experiments. *p < 0.05, **p < 0.01, ***p < 0.001, P > 0.05 labeled as N.S was considered no significance

The construction of CA125 CAR and CR elements is shown in Fig. 1A. The scFv and mesothelin fragments could bind to the CA125-coding DNA sequences (Additional file 8: Table S1), which are connected to the CD8 signal peptide, CD8 hinge region, and CD8 transmembrane coding DNA sequence as the extracellular signal region. The extracellular signal region was concatenated with the intracellular signal region comprising 4-1BB and CD3ζ to form the complete CAR and CR structures, respectively, which were constructed into an RNA-expressing plasmid for in vitro transcription (Fig. 1B). T cells were activated and expanded in vitro for approximately 15–20 days in the resting state (Additional file 1: Fig. S1A, B). Harvested CR and CAR mRNA was individually or simultaneously electroporated into resting T cells. After 4 h, the positive ratio of CAR expression to CR expression on the T cell surface was determined using Fc-labeled CA125 full-length proteins. Flow cytometry staining showed that T cells in the CAR, CR, and CR + CAR groups could combine with the CA125 protein, with a binding efficiency of over 90%. Thus, CR- and CAR-expressing T cells could recognize CA125 without binding site competition (Fig. 1C). Notably, we used diluted CA125 protein to define the affinity of our constructs. We found that the affinity of either CR or CAR was in the picomolar range, which suggests that the binding abilities of mesothelin and CA125 were adequately strong for cellular signal transduction [25]. However, we also observed that the affinity of CR is 5–sixfold lower than that of CAR and approximately twofold lower than that of CR + CAR. Thus, compared with CAR, CR + CAR fine-tuned T cell affinity to CA125 (Fig. 1D; Additional file 2: Fig. S2). To determine the persistence of our constructs in T cells, we also monitored the MFI value in each group after electroporation and found that CAR and CR were still detectable for 9 days (Additional file 3: Fig. S3). Specific primers were then designed (Additional file 8: Table S1) and verified by real-time quantitative PCR to detect CR and CAR elements to confirm whether the plasmids were successfully transduced into cells. Compared with cells in the non-transduced group (NTD), CR-T cells specifically expressed the mesothelin fragment, whereas CAR-T cells specifically expressed the 4H11 scFv fragment. In addition, compared with CR- or CAR-expressing T cells, CR + CAR-T cells expressed an equivalent level of mesothelin and scFv fragments (Fig. 1E). Collectively, we demonstrated the successful generation of T cells expressing CR and CAR.

### CA125-targeting CAR- and CR-co-expressing T cells synergistically enhance tumor clearance toward ovarian cancer cells

**Fig. 2:**
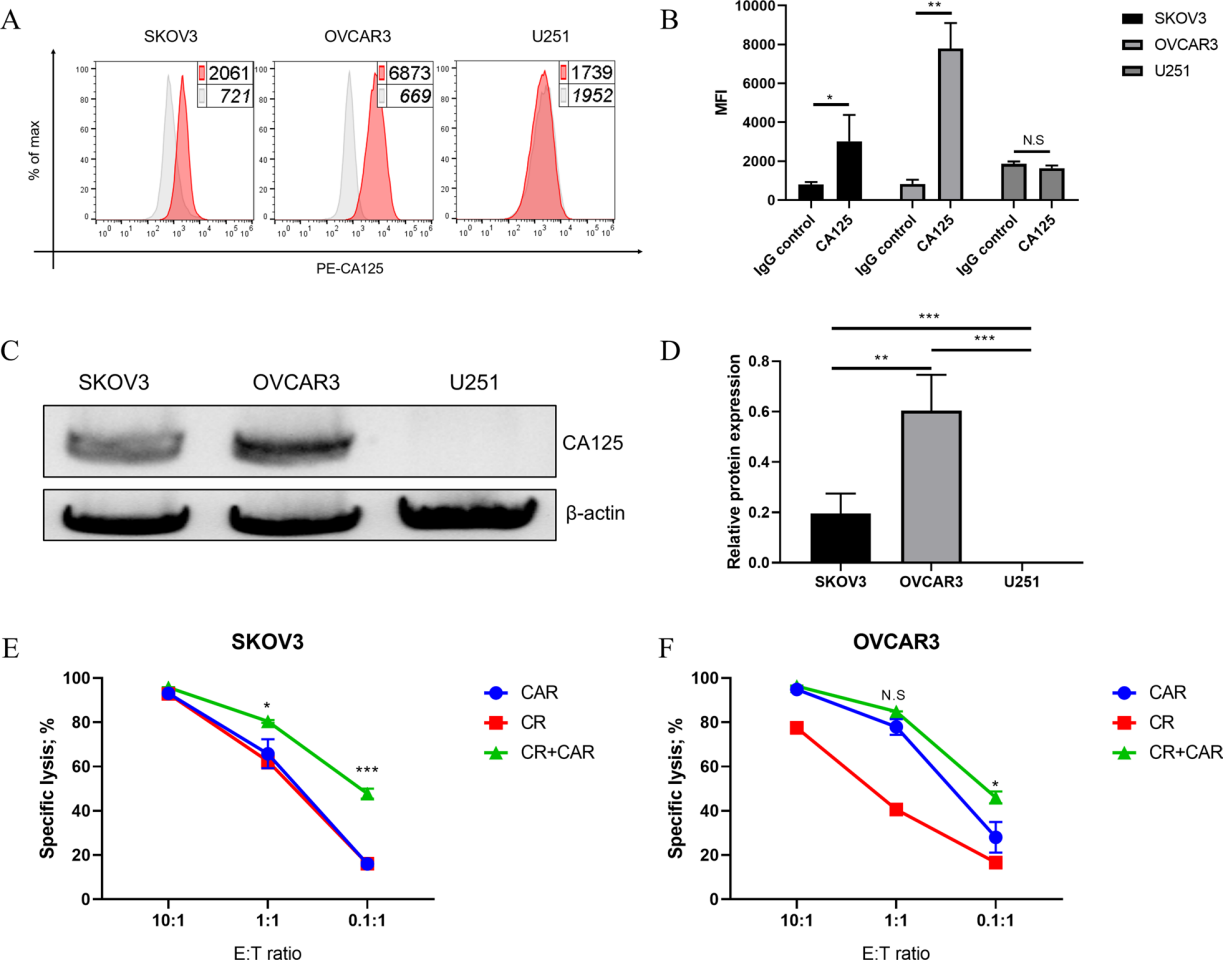
CA125 expression in tumors and killing assay. **A** Flow cytometry to detect the expression of CA125 on the surface of SKOV3, OVCAR3, and U251 cells; **B** summary of CA125 staining MFI of SKOV3, OVCAR3, and U251 cells; **C** western blot to detect the protein expression of CA125 in SKOV3, OVCAR3, and U251 cells; **D** statistic of CA125 expression detected using western blot. **E** Detection of the killing ability of CR, CAR, and CR + CAR expressed T cells targeting SKOV3. **F** Detection of the killing ability of CR, CAR, and CR + CAR expressed T cells targeting OVCAR3. All data are presented as the mean ± SEM of triplicate experiments. *p < 0.05, **p < 0.01, ***p < 0.001, P > 0.05 labeled as N.S was considered no significance

Both ovarian cancer cell lines SKOV3 and OVCAR3 expressed CA125 at high levels, whereas U251 cells showed low CA125 expression undetectable by FACS staining. Thus, U251 cells could be used as a negative control (Fig. 2A, B). Additionally, we assessed CA125 expression in ovarian cancer cells using western blotting. CA125 expression in OVCAR3 cells was slightly higher than that in SKOV3 cells, whereas U251 cells did not express CA125 (Fig. 2C, D). To evaluate the in vitro tumor clearance ability of our engineered T cells, we stably expressed luciferase-GFP in three tumor cell lines by lentiviral transduction. The cells were co-cultured with our engineered T cells independently at a gradient effector/target ratio. The killing potential of different groups of T cells on tumor cells was observed for 72 h using an Incucyte real-time dynamic cell imaging analysis system. The killing efficiencies of CAR-T and CR-T cells were approximately the same as those of the targeted SKOV3 cells. However, the killing efficiencies of CAR- and CR-co-expressing T cells were significantly elevated, especially at E/T ratios of 1:1 or 0.1:1 (Fig. 2E). When it came to OVCAR3 cells, the killing efficiency of CAR-T cells was significantly higher than that of CR-T cells, whereas the killing efficiency of CAR- and CR-co-expressing T cells was significantly higher than that of other groups at an E/T ratio of 0.1:1 (Fig. 2F). Not all engineered T cells made by us exerted a significant killing effect on the U251 cell line (Additional file 4: Fig. S4). These results indicate that CR may not fully supersede CAR in tumor killing, but CAR- and CR-co-expressing T cells exhibit a significantly enhanced ability to target ovarian cancer, which indicates that CR and CAR exert a synergistic effect on CA125-expressing ovarian cancer cells.

### The co-expression of CA125-targeting CAR and CR induces superior T-cell activation and cytotoxicity

**Fig. 3:**
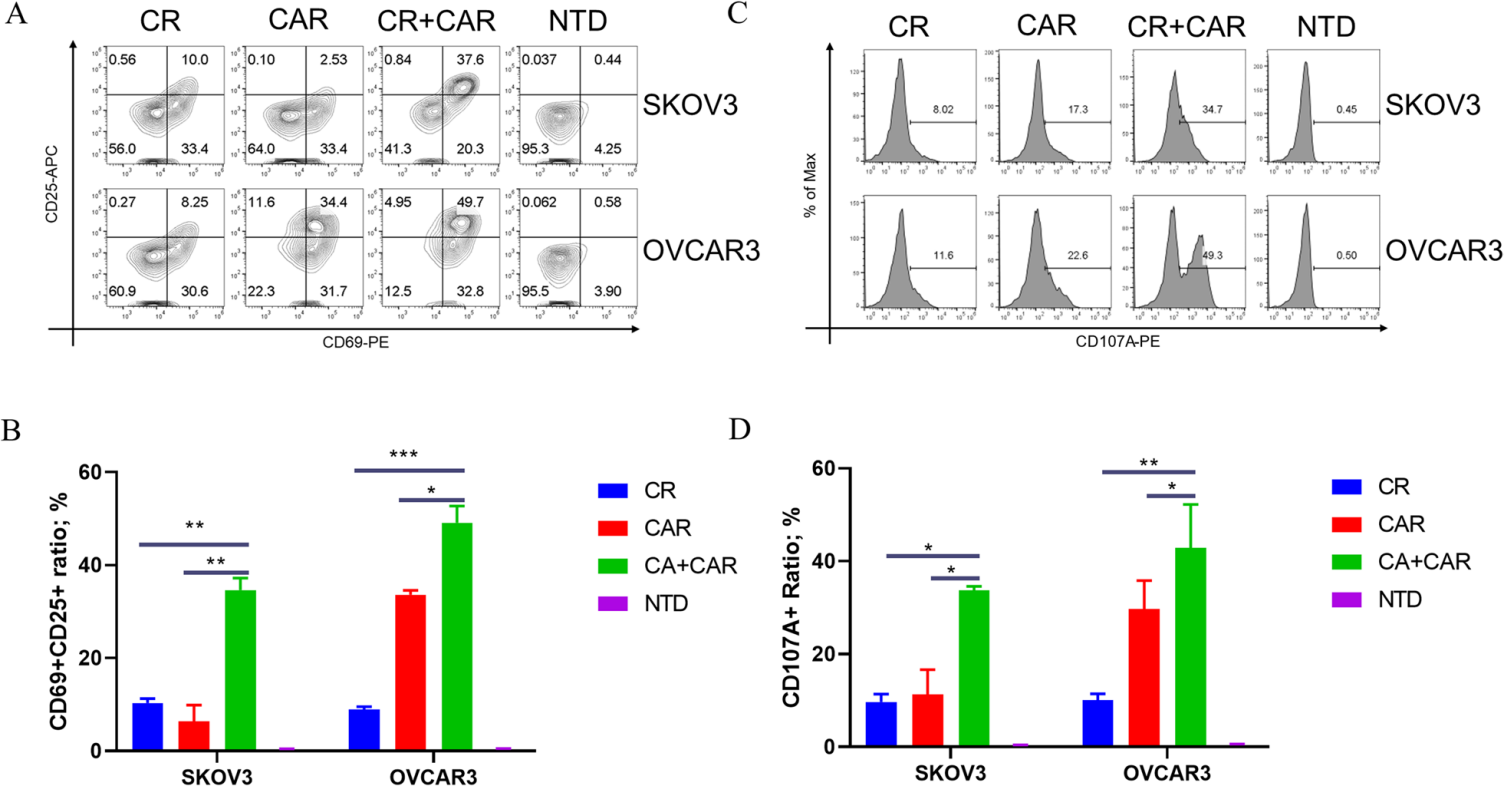
Activation marker detection in our engineered T cells co-cultured with tumor cells. **A** Flow cytometry detection of CD69 and CD25 expression in CR, CAR, CR + CAR, and NTD groups after co-culture of T cells with ovarian cancer cells SKOV3 and OVCAR3 for 24 h. **B** Statistical chart of the proportion of CD69 and CD25 double-positive T cells in each group. **C** Flow cytometry detection of CD107A expression in CR, CAR, CR + CAR, and NTD groups after co-culture of T cells with ovarian cancer cells SKOV3 and OVCAR3 for 4 h; **D** statistical chart of CD107A positive population in T cells of each group. All data are presented as the mean ± SEM of triplicate experiments. *p < 0.05, **p < 0.01, ***p < 0.001, P > 0.05 labeled as N.S was considered no significance

During T-cell activation, CD69 and CD25 are expressed on the T-cell surface [26]. Therefore, we speculated that CAR- and CR-co-expressing T cells synergistically contribute to the activation of T cells. After the co-culture of engineered T cells with tumor cells for 24 h, we measured the expression levels of CD69 and CD25 in T cells. Compared with those in the NTD group, the CD69 + CD25 + cell populations in the CR- or CAR-expressing T cell groups were moderately upregulated, whereas the CD69 + CD25 + cell population in the CR + CAR-expressing T cell group was much larger than that in the CR or CAR groups. The results showed that the CR- and CAR-co-expressing cells induced greater T-cell activation than CAR or CR alone (Fig. 3A, B). Similarly, CD107A, the most sensitive molecular marker for T cell-killing function, has been widely used in functional studies on NK and CD8+ T cells [27]. After co-culture with tumor cells for 4 h, the expression of CD107A in each engineered CD8+ T cell was detected by Golgi blocker treatment [28]. The combination of CR and CAR increased the expression level of CD107A in CD8+ T cells compared with that in the CR or CAR group (Fig. 3C, D). However, we did not observe the activation of CD69, CD25, or CD107A after the co-culture of our engineered T cells with U251 cells (Additional file 5: Fig. S5). In addition, we co-cultured T cell with tumor at E:T ratio = 1:5 in order to imitate the harsh in-vivo environment, we found the proliferation of CR- and CAR-expressing cells is significantly enhanced compared with that of CR- or CAR-expressing cells after co-culture with ovarian cancer cells. Meanwhile, the proliferation of NTD cells gradually decreased during culturing (Additional file 6: Fig. S6). This evidence collectively demonstrated that CA125-targeting CAR- and CR-co-expressing T cells exhibit superior activation and cytotoxicity toward ovarian cancer cells.

### CA125-targeting CAR- and CR-co-expressing cells significantly enhanced IL-2 and IFN-γ secretion by T cells

**Fig. 4:**
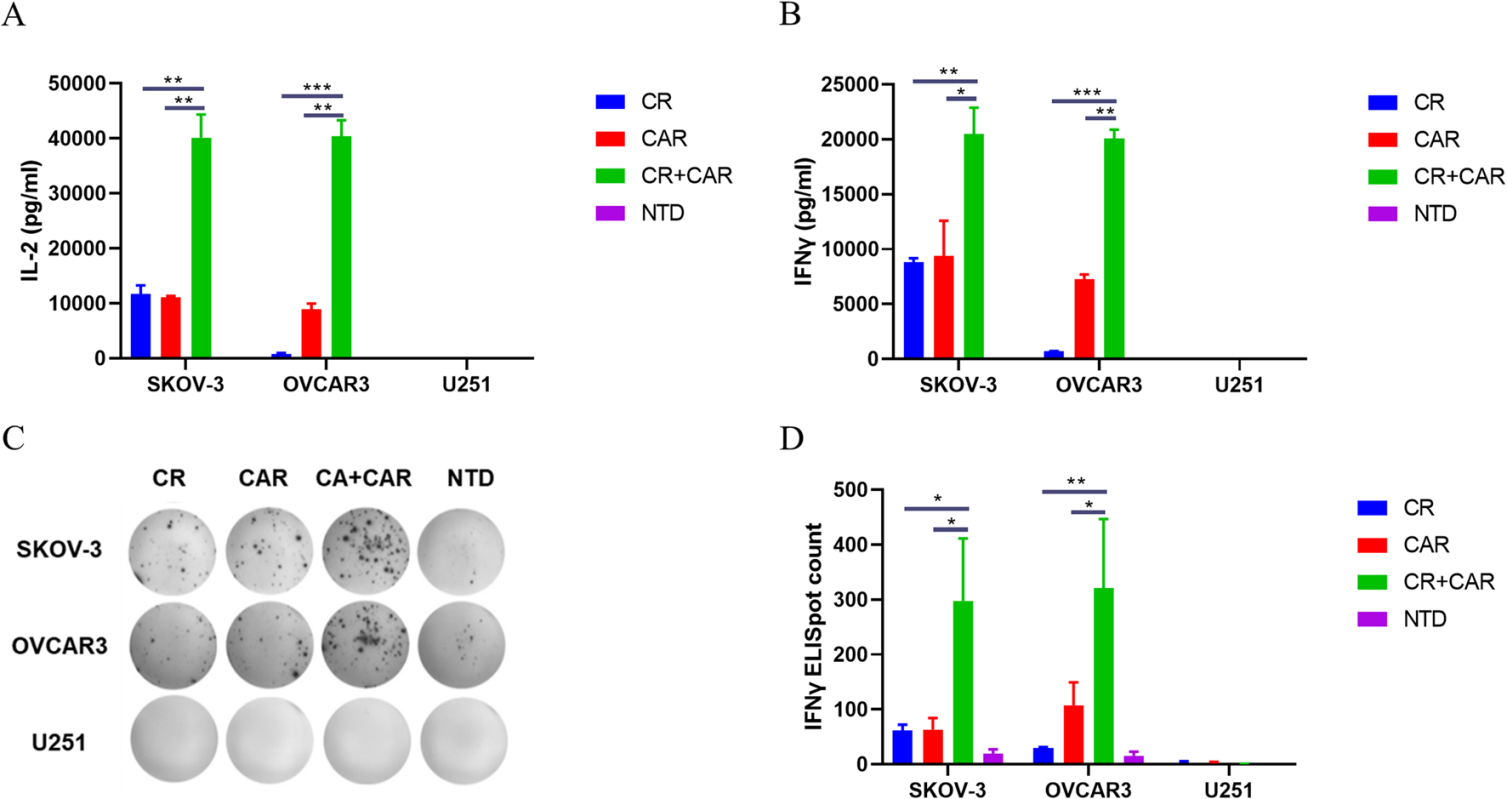
Cytokine secretion of activated engineered T cells. **A** ELISA detection of IL2 secretion capacity after CR, CAR, CR + CAR, and NTD T cells co-cultured with tumor cells for 24 h; **B** ELISA detection of IFN after 24 h of co-culture between T cells and tumor cells in each group—Secretory capacity of gamma. **C** Diluted ELISpot detection of IFN-γ secretion capacity after co-culture of T cells and tumor cells in each group for 24 h; **D** statistical graph of the number of IFNγ spots in each group in ELISpot experiment. All data are presented as the mean ± SEM of triplicate experiments. *p < 0.05, **p < 0.01, ***p < 0.001, P > 0.05 labeled as N.S was considered no significance

Activated T cells produce IL-2 to promote the proliferation, differentiation, and survival of T cells and simultaneously secrete the pro-inflammatory cytokine IFN-γ, which helps transform T cells into cytotoxic T cells. The level of these two factors is closely related to the efficacy of CAR-T cell therapy [29]. Therefore, after the cells were co-cultured with tumor cells for 24 h, we simultaneously investigated the secretion potential of IL-2 and IFN-γ by our engineered T cells in each group using ELISA (Fig. 4A, B). Consistent with the findings of the activation and cytotoxicity assays, the release of IL-2 and IFN-γ from cells in the CR + CAR-T group was significantly enhanced compared with that in cells from the CR or CAR-T groups targeting both SKOV3 and OVCAR3 cells. In contrast, when the cells were co-cultured with U251 cells, IL-2 and IFN-γ secretion in each group of cells was undetectable. To validate this phenomenon, we conducted a diluted ELISpot experiment to determine the secretion potential of IFN-γ in each engineered T cell. Similarly, we detected a few IFN-γ spots in the CR or CAR-T groups, but the number of IFN-γ spots in the CR + CAR group was significantly greater (Fig. 4C, D). The above findings show that T cells expressing both CR and CAR had a significantly stronger antitumor function.

### The co-expression of CA125-targeting CAR and CR enhances the in vivo killing efficacy of T cells against ovarian cancer

**Fig. 5:**
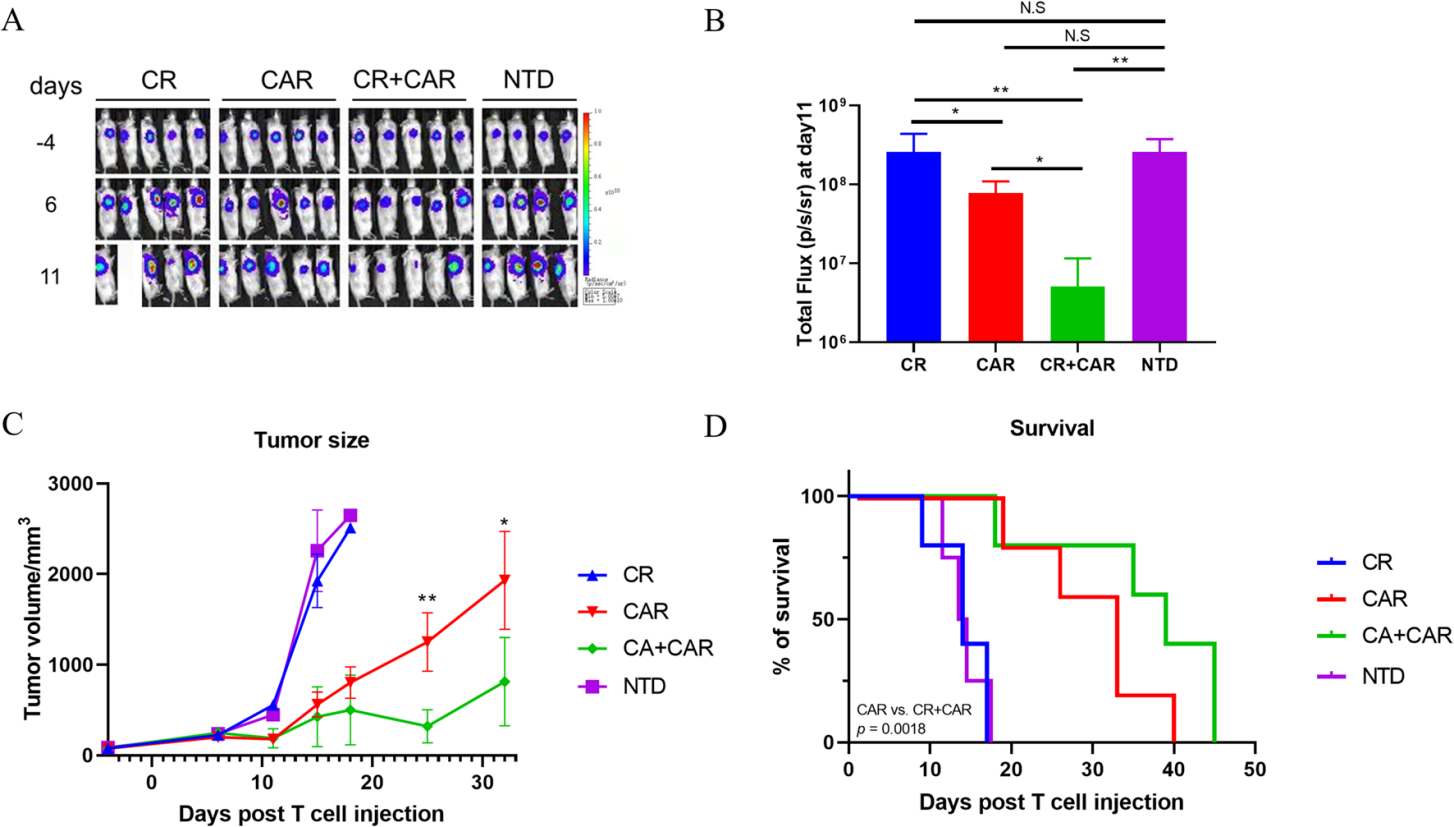
The effect of engineered T cells targeting ovarian cancer cell line in vivo. **A** Representative bioluminescence images of tumor killing capacity of engineered T cells in NSD/SCID mice with SKOV3-LucG xenograft model. **B** Quantification of bioluminescence kinetics of SKOV3-LucG tumor growth in the xenograft model after T cell injection at day 11. **C** The tumor size of each group was measured via a Vernier caliper. **D** The Kaplan–Meier survival curve of mice in the SKOV3-LucG xenograft model. Mice were sacrificed when the tumor volume reached 2000 mm^3^. All data are presented as the mean ± SEM of triplicate experiments. *p < 0.05, **p < 0.01, ***p < 0.001, P > 0.05 labeled as N.S was considered no significance

To evaluate the antitumor effects of our engineered T cells in vivo, SKOV3-LucG tumor cells were subcutaneously implanted into NSG mice. Fourteen days later, the mice were intravenously injected with the engineered T cells. Luminescence imaging results showed that the tumor gradually shrunk in the CAR and CR + CAR groups, whereas tumor control in the CR group did not show any advantage over that in the NTD group (Fig. 5A). However, the CR + CAR group had a greater tumor clearance rate than the CAR group on day 11, as indicated by imaging statistics (Fig. 5B). We also observed that only CAR-expressing T cells and CR + CAR expressing T cells could effectively control SKOV3 tumor cell growth after 30 days, as indicated by the tumor size measurement results. Meanwhile, the tumors kept expanding in mice treated with NTD or CR-expressing T cells, and the mice were sacrificed in 15 to 20 days (Fig. 5C). Furthermore, CR + CAR + CAR-expressing T cells showed obvious survival advantages over other groups (Fig. 5D). In summary, we demonstrated that, compared with CR- or CAR-expressing T cells or NTD cells. CR + CAR-expressing T cells showed prominent antitumor properties in ovarian cancer in vivo models and increased mouse survival.

### The effect of CR combined with CAR on T-cell signaling

**Fig. 6:**
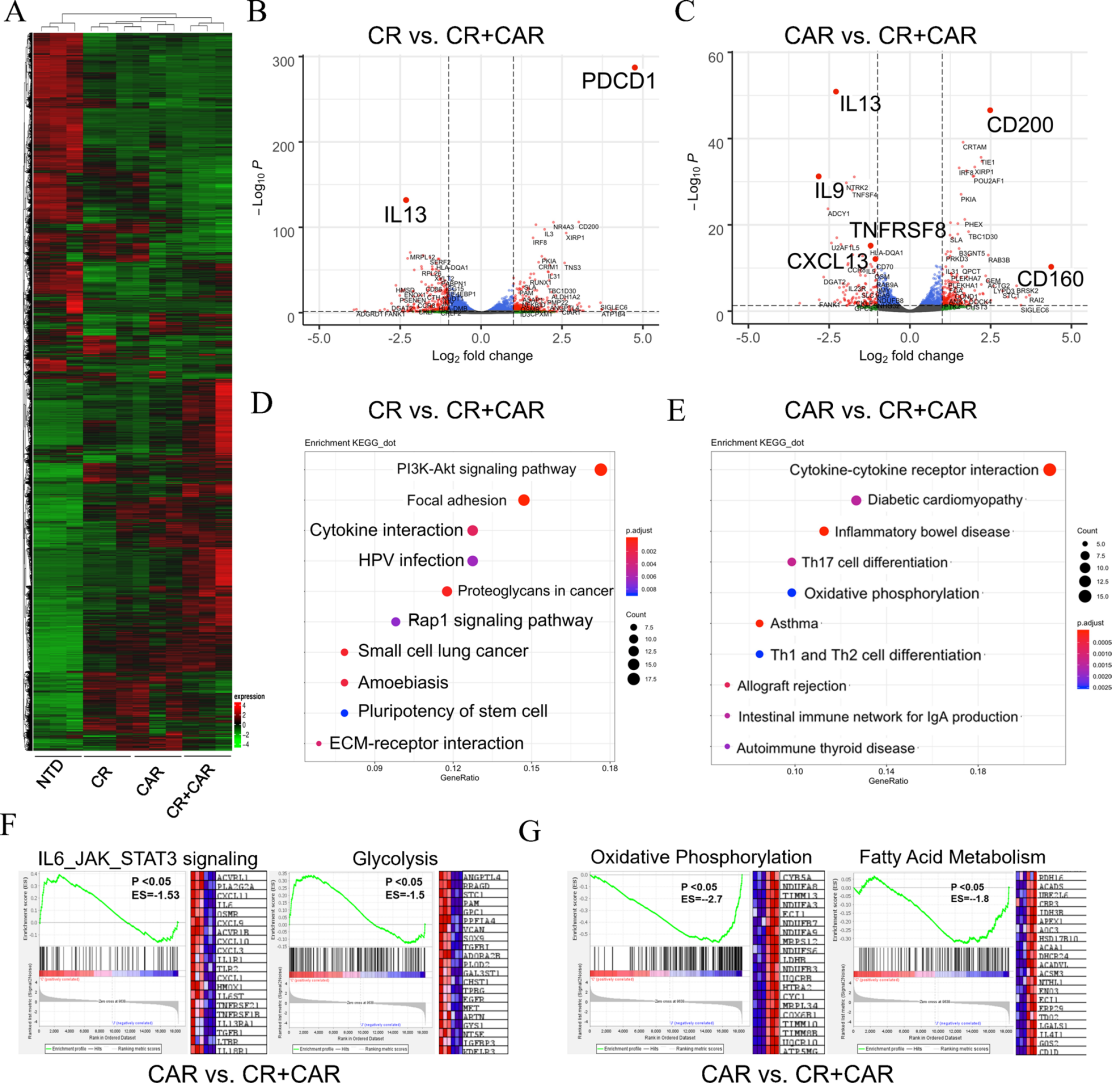
Transcriptional signatures and functional properties of our engineered T cells. **A** Heat map of differences in gene expression in the whole transcriptome after 24 h of co-culture of CR, CAR, CR + CAR, and NTD T cells and tumor cells. **B** Volcano plots show differences in transcriptional gene expression between CR and CR + CAR group T cells and tumor cells after 24 h of co-culture. **C** Volcano plot of volcanic differences in transcriptional gene expression between T cells in the CAR and CR + CAR groups and tumor cells after 24 h of co-culture; **D** KEGG pathway analysis of CR and CR + CAR group T cells expression difference gene mainly involved in the signaling pathway; **E** KEGG pathway analyzes the signaling pathways in which T cells in the CAR and CR + CAR groups express differences in genes. **F** Gene set enrichment analysis of IL6/JAK/STAT3 and Glycolysis signaling gene sets in CAR and CR + CAR T cells from RNA-seq, the heatmap of the top 20 up-regulated genes in the CAR group was listed in each gene set. **G** Gene set enrichment analysis of Oxidative Phosphorylation and Fatty Acid Metabolism signaling gene sets in CAR and CR + CAR T cells from RNA-seq, the heatmap of the top 20 down-regulated genes in the CAR group was listed in each gene set. (n = 3 biological replicates). All data are presented as the mean ± SEM of triplicate experiments. *p < 0.05, **p < 0.01, ***p < 0.001, P > 0.05 labeled as N.S was considered no significance

To further characterize each engineered T cell, we co-cultured CR-, CAR-, or CR + CAR-expressing T cells with SKOV3 cells for 24 h. After T-cell separation and purification, stimulated T cells from each group were isolated for transcriptome sequencing analysis. The gene expression in each group was significantly separated by hierarchical clustering analysis of the expression profiles (Fig. 6A). Notably, 537 genes were upregulated and 614 genes were downregulated in the CR group compared to that in the CR + CAR group (Fig. 6B). PD-1 mRNA expression was elevated in the CR group, whereas PD-1 protein expression was lower in the CR group than in the CAR or CR + CAR groups (Additional file 7: Fig. S7A, B). Thus, we hypothesized that PD-1 transcription in each group may exhibit spatial and temporal disparities. Therefore, we assessed PD-1 mRNA expression at each time point after co-culture with SKOV3 cells and found that PD-1 expression in the CR group was delayed compared with that in the CAR and CR + CAR groups (Additional file 7: Fig. S7C). This indicates that CR may delay T-cell activation and prevent overactivation-induced T-cell exhaustion [30]. Compared with that in the CAR group, 493 genes were upregulated and 725 genes were downregulated in the CR + CAR group, and immune-regulator genes, including IL-9, IL-13, BATF3, and CXCL13, and several TNFRSF proteins were significantly downregulated. This phenomenon was associated with T cell homing and memory phenotype persistence [31–33]. We speculated that CR naturally binding to CA125 can offer suitable T cell differentiation signals to promote T-cell survival and persistence, whereas CAR stimulation can offer T-cell activation signals to promote T cell cytotoxic function (Fig. 6C). Next, we performed differential gene signaling pathway enrichment analysis and found that the PI3K/AKT signaling pathway, cell adhesion, and cytokine receptor pathways were significantly altered in the CR group compared with that in the CR + CAR group (Fig. 6D). Cell adhesion, Th17 cell differentiation, and oxidative phosphorylation were significantly altered between the CAR and CR + CAR groups (Fig. 6E). GSEA showed that the glycolytic pathways and IL-6/JAK/STAT3 pathways were elevated. Oxidative phosphorylation (OP) was also elevated (Fig. 6F), and fatty acid metabolism (FAM) was suppressed in the CAR group compared with that in the CR + CAR group (Fig. 6G). As OP and FAM are associated with mitochondrial fitness [34], we confirmed that CR + CAR activation could synergistically promote T cell persistence and function. In conclusion, these results demonstrated that T cells in the CR + CAR group had significantly altered gene expression profiles and signaling pathways, which primarily contribute to the activation and cytotoxicity of T cells targeting CA125.

## Discussion

With the rapid development of genetic engineering methods and in-depth investigations of the molecular mechanism underlying T cell recognition, scientists have developed chimeric antigen receptors (CARs), which endow T cells with the ability to recognize tumor antigens in an HLA-independent manner and enable them to recognize more extensive target antigens than natural T-cell surface receptors [35]. However, the treatment of solid tumors with CAR-T cells has multiple obstacles. On one hand, scFv, as a synthetic antibody, leads to the internalization and degradation of tumor antigens in the course of treatment, leading to treatment failure [36]; however, owing to the complex tumor microenvironment and heterogeneity of solid tumors, conventional T cells cannot be activated at the tumor site, which leads to the escape of tumor cells [37]. In ovarian cancer, the interaction between mesothelin and CA125 promotes the metastasis of ovarian cancer cells and stromal tumors, indicating that this combination has a tumor-promoting effect. In this study, we designed a new type of chimeric receptor T cell targeting CA125, based on the natural binding of mesothelin to CA125 in patients with ovarian cancer, to reverse the role of this ligand-receptor binding activity in promoting tumor transformation. In addition, because stably expressed CAR-T cells may cause on-target-off tumor effects that can be life-threatening [38], we transiently expressed CR and CAR in T cells as a safety consideration.

Our findings show that the therapeutic CA125-targeting effect of CR T cells is similar to that of CAR T cells, but it cannot eliminate tumor cells. However, CR- and CAR-co-expressing T cells targeting CA125 showed prominent antitumor activity, comprising the expression of cell activation markers CD69 and CD25 and the early activation marker CD107A. CR or CAR-T cells can bind to CA125 but cannot manipulate cell function in a biological environment. This may be because CR or CAR binding to CA125 is not stable. The extracellular segment of CA125 comprises 156 amino acids forming a glycosylation repeat sequence, which are easy to hydrolyze [39]. This leads to the secretion of CA125 into the serum and body fluid, resulting in insufficient activation signals. However, when CR and CAR are co-expressed on T cells, the therapeutic effect of T cells can be significantly improved by IL-2 and IFN-γ secretion, suggesting that CR-T cells may reduce the resistance of tumor cells during the treatment process to help CR and CAR prolong the timing of CA125 interaction. In addition, the superior antitumor effect of CR- and CAR-expressing T cells was also demonstrated in vivo.

Through transcriptome sequencing, we also demonstrated that the combination of CR and CAR has a significant impact on the expression levels of multiple immune-related genes and signaling pathways during T-cell activation. Notably, the upregulation of IL-13 was most significant in CR- and CAR-co-expressing T cells compared with that in CR- or CAR-expressing T cells. IL-13 was an immunoregulatory cytokine produced primarily by Th2 cells that may improve T cell regulation and prevent T cell overreaction [40, 41]. In addition, IL-9 [31], BATF3 [32], and CXCR13 [33], associated with T cell homing and memory phenotype persistence, were also upregulated in the CR + CAR group compared with that in the CAR group, indicating that CR interaction could reverse CAR-induced T cell overactivation and fine-tune T cells in a healthy state. Moreover, GSEA showed enhanced mitochondrial fitness [42] in activated CR- and CAR-expressing T cells. Both findings confirm that CR is functionally beneficial for T cells.

In summary, our data highlight a novel T cell engineering strategy that combines the ligand-receptor motif and antibody-antigen motif to enhance tumor reactions and optimize T cell phenotypes, providing translational potential for enhancing CAR T cell therapeutic efficacy in ovarian cancers.

### Supplementary Information

**Additional file 1: Figure S1**. T cell growth and size during in vitro culturing, A statistical analysis was conducted on the number of T cells from two individuals over a 20-day period of in vitro culturing, with T cell stimulation occurring on day 1. B. Statistical analysis was performed on the size of T cells from these same individuals during 20 days.

**Additional file 2: Figure S2.** T cell affinity assay. A. FASC plot of different concentration of CA125 staining in each CAR, CR and CR + CAR group with half NTD cells as negative control. B. statistical analysis of affinity of CAR, CR and CR + CAR using method from patent (*CN 111351936 A*).

**Additional file 3: Figure S3.** FACS assay of CAR, CR and CR + CAR point-in-time expression until 9 days after electroporation.

**Additional file 4: Figure S4**. Incucyte killing assay of CAR, CR and CR + CAR expressed T cells after co-cultured with each SKOV3, OVCAR3 and U251 cell line at different E:T ratio.

**Additional file 5: Figure S5.** Activation marker detection in CAR, CR and CR + CAR expressed T cells and NTD cells co-cultured with U251 cells at E:T = 1:1 after 24 h.

**Additional file 6: Figure S6.** CAR, CR and CR + CAR expressed T cells and NTD cells expansion after co-cultured with SKOV3 cell line at E:T = 1:5.

**Additional file 7: Figure S7.** Dynamic expression of PD-1 in CAR, CR and CR + CAR expressed T cells and NTD cells after co-cultured with SKOV3 cell line at E:T = 1:1. A. FACS plot of PD-1 expression after co-cultured with SKOV3 for 24 h; B. qPCR detection of PD-1 mRNA expression in indicated time-point after co-cultured with SKOV3 during 96 h.

**Additional file 8: Table S1.** The DNA sequence of CR and CAR sturcture and primers for CR and CAR detection.

## Data Availability

The datasets generated and/or analysed during the current study are not publicly available due to further analysis but are available from the corresponding author on reasonable request.

## Abbreviations

OC: Ovarian cancer
CR: Chimeric receptor
CAR: Chimeric antigen receptor
ITAM: Immunoreceptor tyrosine-based activation motif
PBMC: Peripheral blood mononuclear cells
ELISA: Enzyme-linked immunosorbent assay
ELISpot: Enzyme-linked immunospot assay
