# Supplementary figures and images for: Ligand-based adoptive T cell targeting CA125 in ovarian cancer
Source: J Transl Med. 2023 Sep 5;21:596. doi: 10.1186/s12967-023-04271-8 (PMC10481596; doi:10.1186/s12967-023-04271-8)

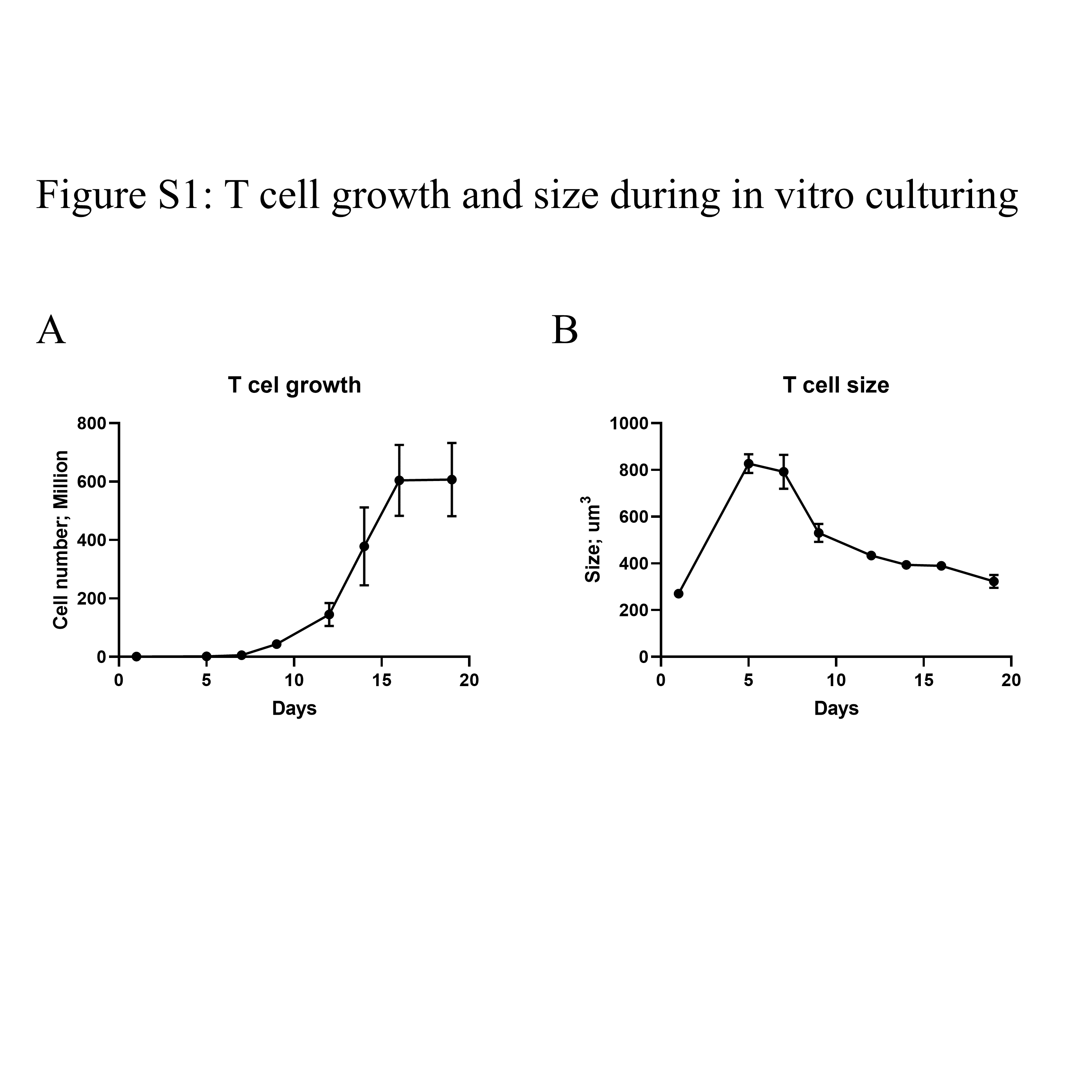

Supplement: Supplementary file 1 — Additional file 1: Figure S1. T cell growth and size during in vitro culturing, A statistical analysis was conducted on the number of T cells from two individuals over a 20-day period of in vitro culturing, with T cell stimulation occurring on day 1. B. Statistical analysis was performed on the size of T cells from these same individuals during 20 days. [file 12967_2023_4271_MOESM1_ESM.png]

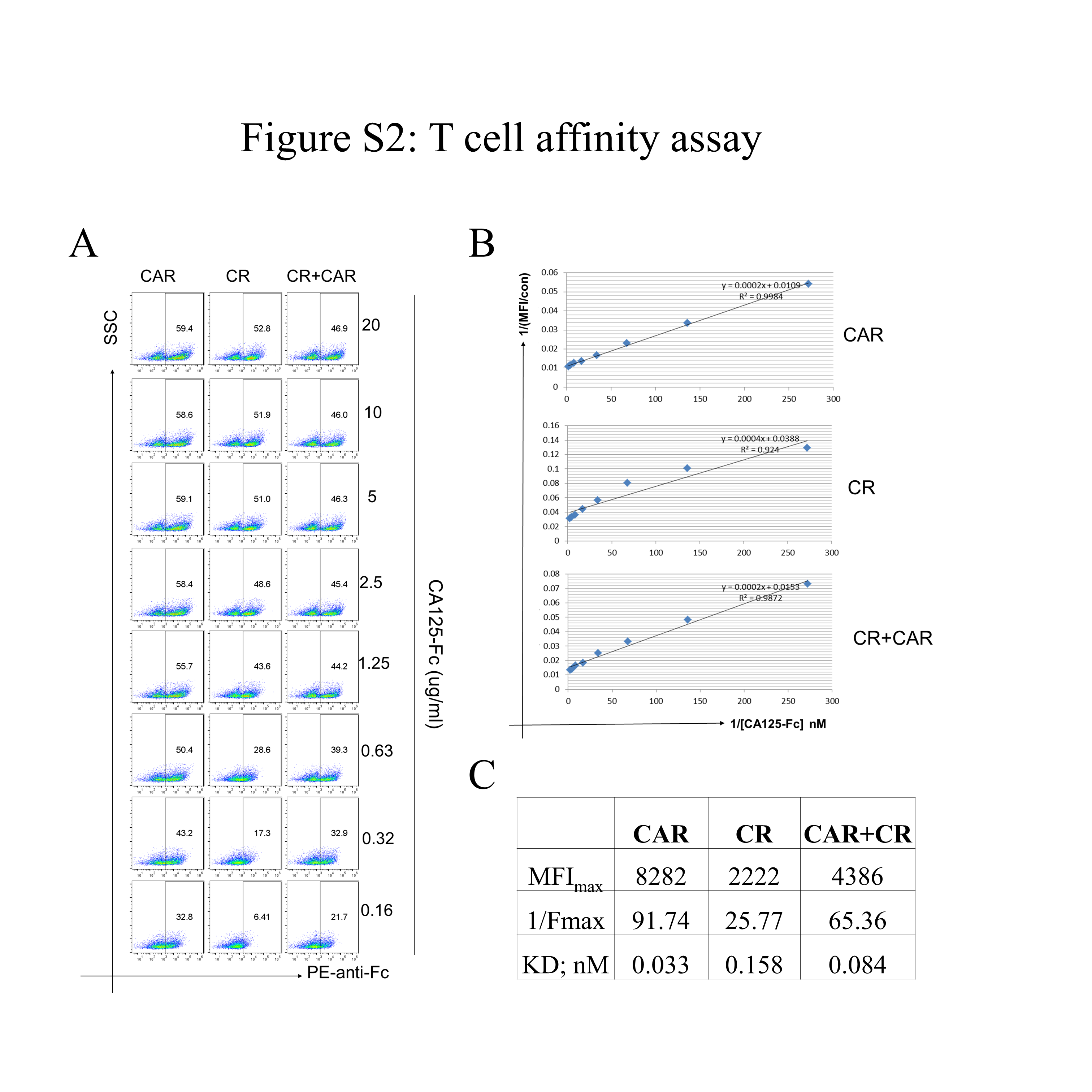

Supplement: Supplementary file 2 — Additional file 2: Figure S2. T cell affinity assay. A. FASC plot of different concentration of CA125 staining in each CAR, CR and CR + CAR group with half NTD cells as negative control. B. statistical analysis of affinity of CAR, CR and CR + CAR using method from patent (CN 111351936 A). [file 12967_2023_4271_MOESM2_ESM.png]

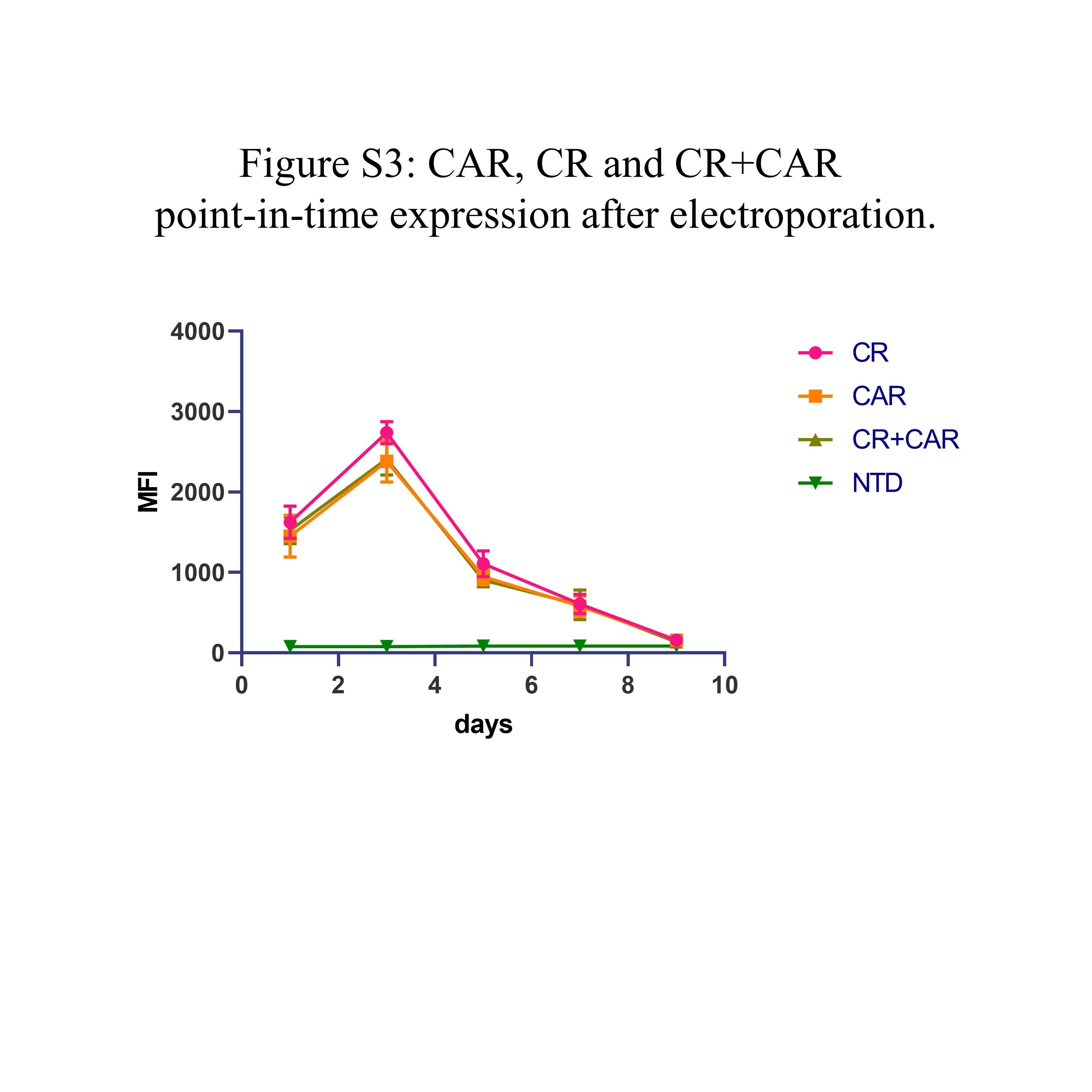

Supplement: Supplementary file 3 — Additional file 3: Figure S3. FACS assay of CAR, CR and CR + CAR point-in-time expression until 9 days after electroporation. [file 12967_2023_4271_MOESM3_ESM.png]

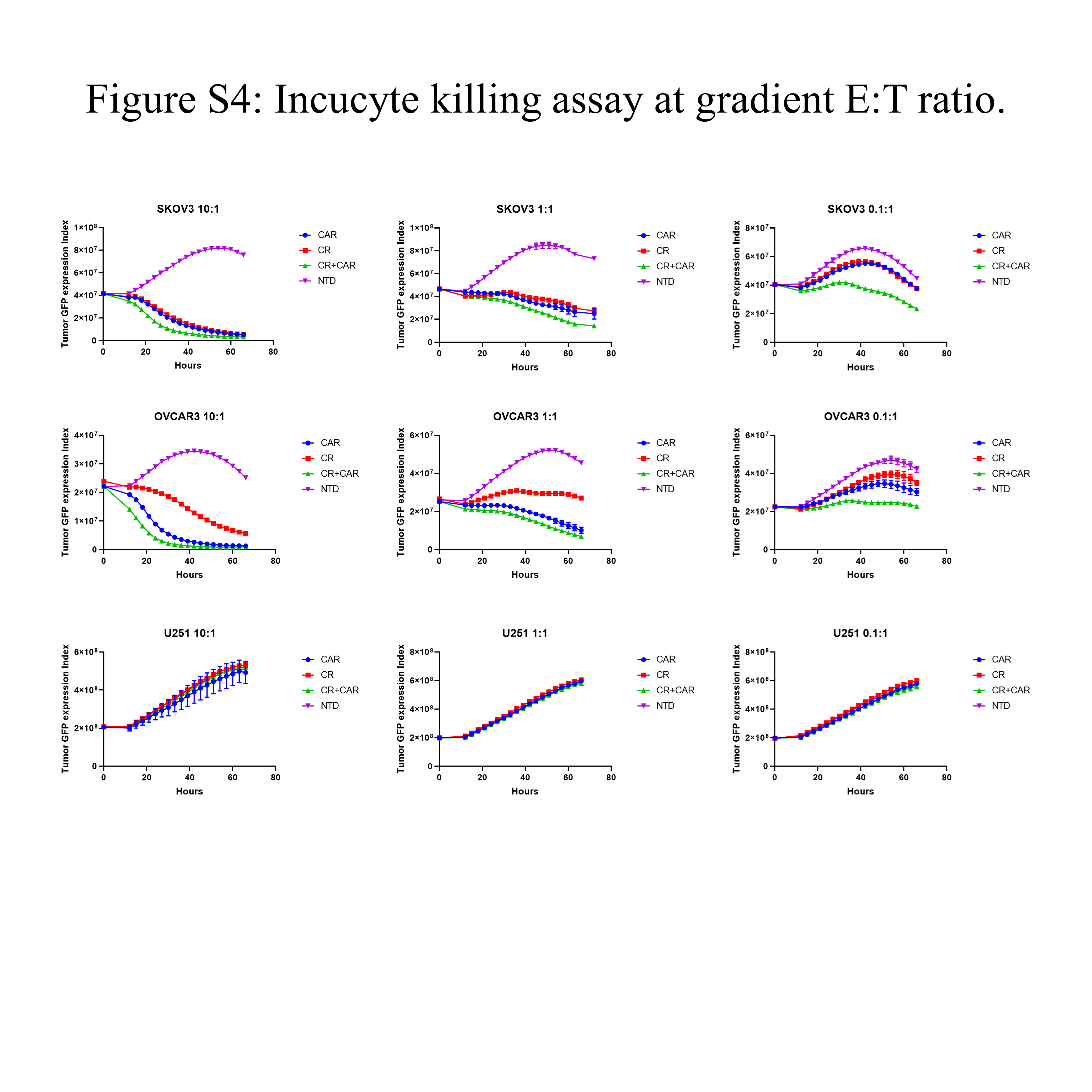

Supplement: Supplementary file 4 — Additional file 4: Figure S4. Incucyte killing assay of CAR, CR and CR + CAR expressed T cells after co-cultured with each SKOV3, OVCAR3 and U251 cell line at different E:T ratio. [file 12967_2023_4271_MOESM4_ESM.png]

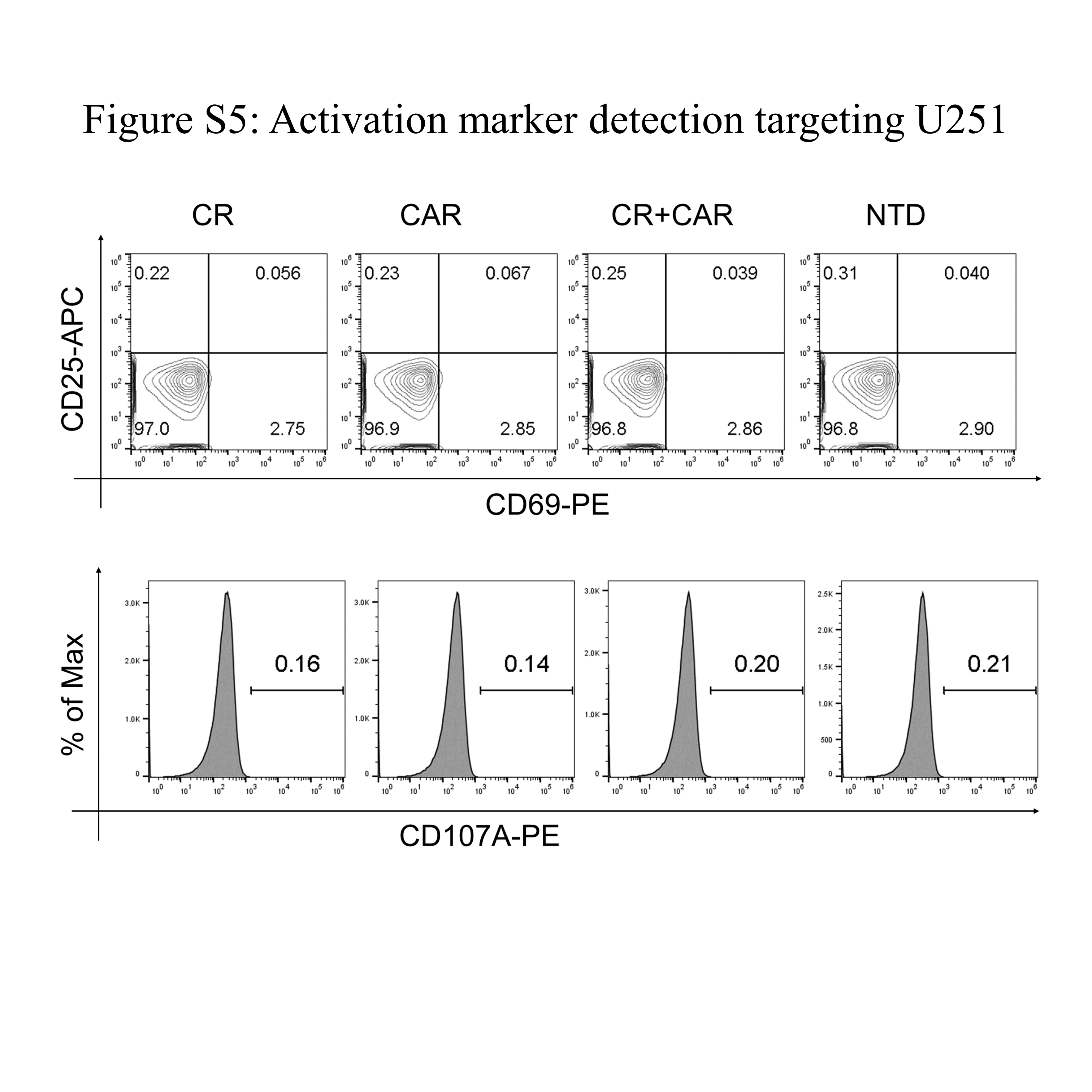

Supplement: Supplementary file 5 — Additional file 5: Figure S5. Activation marker detection in CAR, CR and CR + CAR expressed T cells and NTD cells co-cultured with U251 cells at E:T = 1:1 after 24 h. [file 12967_2023_4271_MOESM5_ESM.png]

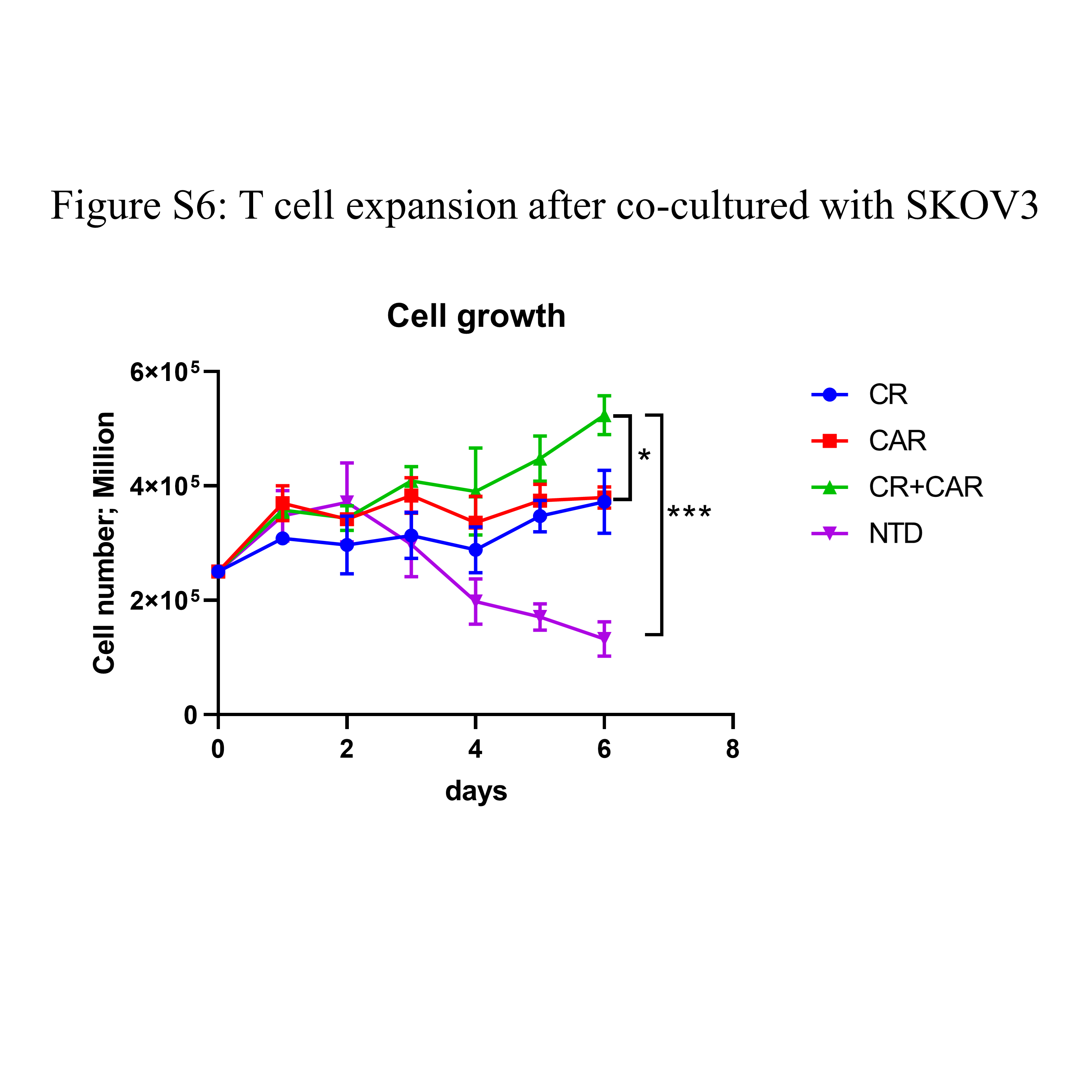

Supplement: Supplementary file 6 — Additional file 6: Figure S6. CAR, CR and CR + CAR expressed T cells and NTD cells expansion after co-cultured with SKOV3 cell line at E:T = 1:5. [file 12967_2023_4271_MOESM6_ESM.png]

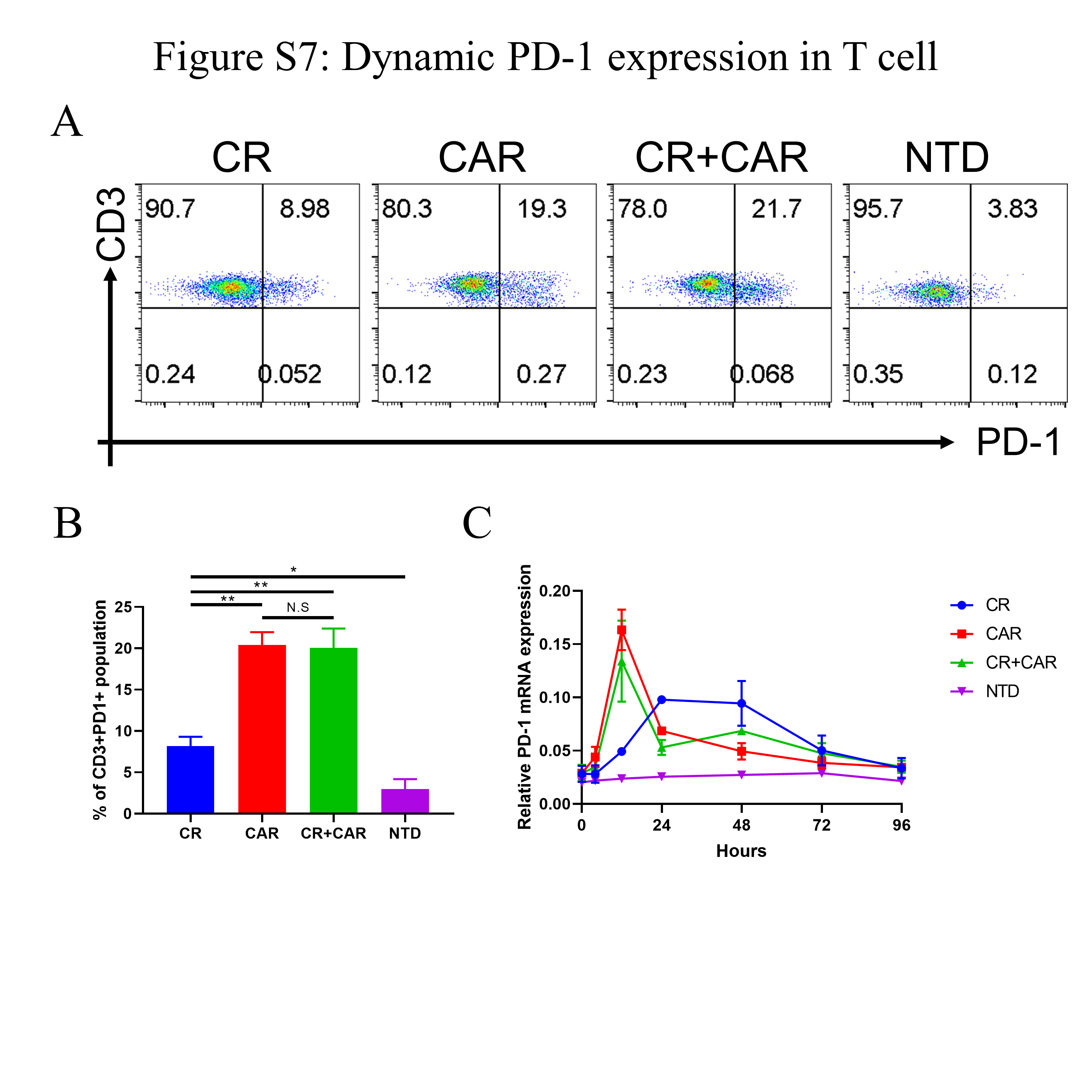

Supplement: Supplementary file 7 — Additional file 7: Figure S7. Dynamic expression of PD-1 in CAR, CR and CR + CAR expressed T cells and NTD cells after co-cultured with SKOV3 cell line at E:T = 1:1. A. FACS plot of PD-1 expression after co-cultured with SKOV3 for 24 h; B. qPCR detection of PD-1 mRNA expression in indicated time-point after co-cultured with SKOV3 during 96 h. [file 12967_2023_4271_MOESM7_ESM.png]
